# Quantitative Proteomics Analysis of Berberine-Treated Colon Cancer Cells Reveals Potential Therapy Targets

**DOI:** 10.3390/biology10030250

**Published:** 2021-03-23

**Authors:** Pengfei Li, Zhifang Hao, Huanhuan Liu, Bojing Zhu, Liuyi Dang, Chen Ma, Yintai Xu, Yiyan Zhang, Daidi Fan, Shisheng Sun

**Affiliations:** 1 College of Life Sciences, Northwest University, Xi’an 710069, China; pengfeilibio@163.com (P.L.); zhifang_hao@163.com (Z.H.); Huanhuan_Liu5@163.com (H.L.); bojingzhu@foxmail.com (B.Z.); liuyi.dang@outlook.com (L.D.); chenmaGlyco@163.com (C.M.); yintaixu@stumail.nwu.edu.cn (Y.X.); yiyan_Cheung@163.com (Y.Z.); 2 Shaanxi Key Laboratory of Degradable Biomedical Materials, School of Chemical Engineering, Northwest University, Xi’an 710069, China; fandaidi@nwu.edu.cn

**Keywords:** berberine, colon cancer, proteomics, potential targets

## Abstract

**Simple Summary:**

Colon cancer is one of the most common malignant tumors and beberine has been found to exert potential anti-colon cancer activity in vitro and in vivo. In this study, by using proteomics and bioinformatics approaches, we report that berberine may inhibit the proliferation of colon cancer cells by regulating mitochondrial translation and ribosome biogenesis, as well as by promoting calcium mobilization and metabolism of fat-soluble vitamins. Moreover, GTPase ERAL1 and mitochondrial ribosomal proteins MRPL11, 15, 30, 37, 40, and 52 have great potential to serve as potential therapeutic targets for colon cancer treatment.

**Abstract:**

Colon cancer is one of the most lethal malignancies worldwide. Berberine has been found to exert potential anti-colon cancer activity in vitro and in vivo, although the detailed regulatory mechanism is still unclear. This study aims to identify the underlying crucial proteins and regulatory networks associated with berberine treatment of colon cancer by using proteomics as well as publicly available transcriptomics and tissue array data. Proteome profiling of berberine-treated colon cancer cells demonstrated that among 5130 identified proteins, the expression of 865 and 675 proteins were changed in berberine-treated HCT116 and DLD1 cells, respectively. Moreover, 54 differently expressed proteins that overlapped in both cell lines were mainly involved in mitochondrial protein synthesis, calcium mobilization, and metabolism of fat-soluble vitamins. Finally, GTPase ERAL1 and mitochondrial ribosomal proteins including MRPL11, 15, 30, 37, 40, and 52 were identified as hub proteins of berberine-treated colon cancer cells. These proteins have higher transcriptional and translational levels in colon tumor samples than that of colon normal samples, and were significantly down-regulated in berberine-treated colon cancer cells. Genetic dependency analysis showed that silencing the gene expression of seven hub proteins could inhibit the proliferation of colon cancer cells. This study sheds a light for elucidating the berberine-related regulatory signaling pathways in colon cancer, and suggests that ERAL1 and several mitochondrial ribosomal proteins might be promising therapeutic targets for colon cancer.

## 1. Introduction

Colon cancer is one of the most common malignant tumors, accompanied with high morbidity and mortality [1]. The incidence is even higher in the most developed countries [2]. With continuing progress in developing countries, the cases of colorectal cancer worldwide are predicted to increase to 2.5 million in 2035 [3]. Surgery, chemotherapy, and radiotherapy are conventional therapies for colon cancer. However, each treatment method is associated with specific adverse effects and complications [4]. In recent years, biologic agents are broadly used to treat colon cancers [5]. Bevacizumab is a humanized monoclonal antibody against vascular endothelial growth factor A (VEGF-A) and is used for the treatment of metastatic colon cancer [6]. Aflibercept is a recombinant fusion protein consisting of VEGF-binding portion from the extracellular domains of human VEGF receptors 1 and 2, and is commonly used as a second-line treatment for metastatic colon cancer [7]. Additionally, ramucirumab as an anti-VEGF receptor 2 monoclonal antibody was approved for targeting angiogenesis for metastatic colon cancer [8]. For KRAS and RAF-wild-type metastatic colon cancer, cetuximab or panitumumab can be used as anti-EGFR agents [9,10]. Despite improvement in the treatment of colon cancer, drug resistance and serious side effects demand specific targeted therapies to reduce toxicity and untoward effects and it is urgent to find a more effective treatment strategy for colon cancer [11].

Berberine is a bioactive isoquinoline derivative alkaloid isolated from Chinese herbs, and is important for the synthesis of several bioactive derivatives by means of condensation, modification, and substitution of functional groups [12,13]. Berberine has been detected, isolated, and quantified from various plant families and genera, and is also widely present in barks, leaves, twigs, rhizomes, roots, and stems of some medicinal plant species [12]. Numerous studies have shown that berberine can be used to treat inflammatory disorders, fevers, cardiovascular diseases, and tumors [14]. A clinical study showed that berberine could prevent recurrent colorectal adenoma with rarely adverse events in clinical practice [15]. Despite its clinical promise, there have been documents revealing its negative impacts on human cells, such as cytotoxicity, genotoxicity, mutagenicity, and carcinogenicity [16]. In addition, there is clinical research that showed that 34.5% of patients with type-2 diabetes that were treated with berberine (500 mg three times/day for 13 weeks) had transient GI side effects of diarrhea, constipation, flatulence, and abdominal complaints [17]. Other research showed that in some patients with refractory cardiac heart failure, ventricular tachycardia after infusion of berberine (0.2 mg/kg/min for 30 min) occurred [18].

Recently, it has been found that berberine also has potential anti-colon cancer effects [19,20]. Gastrointestinal hormones are released by the enteroendocrine cells of the gut [21]. Gastrin as a gastrointestinal hormone has been demonstrated to be a biomarker of cancer risk and a growth factor for colon cancer [22]. One study showed that berberine can significantly reduce gastrin [23], and the data shed a new light into the anti-colon cancer potential of berberine. Moreover, there is a relationship between colon cancer and gut microbiota [24,25]. Modification of gut microflora and administration of probiotics accelerate the healing of the colon injury [26], and may lead to prevention and treatment of colon cancer [24]. Previous studies also showed that berberine could inhibit cell proliferation, invasion, and metastasis, as well as promote cell apoptosis in colon cancer cells [27,28,29]. However, the mechanisms and pathways that mediate the multiple pharmacological actions of berberine have not been fully elucidated [30].

In this study, we comparatively analyzed the protein changes in berberine-treated colon cancer cells using quantitative proteomics, a powerful tool to efficiently evaluate drug action [31]. The differently expressed proteins (DEPs) were clustered into different expression pattern clusters using soft clustering with the concentration gradient. The overlapping DEPs detected in HCT116 and DLD1 cells were further investigated with various bioinformatics approaches, such as Gene Ontology (GO) enrichment, Kyoto Encyclopedia of Genes and Genomes (KEGG) pathway analysis, and Protein–Protein Interaction (PPI) network integration. Moreover, seven hub proteins were pinpointed with the comparison of our proteomics data and as well as another set of proteomics data of berberine-treated colon cancer cells [32]. Finally, we investigated the transcriptional and translational levels of seven hub proteins in colon adenocarcinoma tissue versus normal tissue by Gene Expression Profiling Interactive Analysis (GEPIA) and Human Protein Atlas (HPA), as well as validated their genetic dependencies by Cancer Dependency Map (DepMap) datasets. Our data may provide potential biological candidates for further studying of the mechanisms of berberine for colon cancer treatment.

## 2. Materials and Methods

### 2.1. Cell Culture

The human colon cancer cell lines HCT116, DLD1, SW480, HT29, HCT8, LOVO, and CACO2 were purchased from the American Type Culture Collection (Manassas, VA, USA). Cell lines were cultured in McCoy’s 5A, Dulbecco’s modified eagle media or Roswell Park Memorial Institute-1640 medium (Hyclone, Logan, UT, USA) supplemented with 10% fetal bovine serum (Bioind, Haifa, Israel) and 1% penicillin/streptomycin (Solarbio, Peking, China). All cells were incubated in 5% CO_2_ at 37 °C and were tested for mycoplasma contamination before experiments.

### 2.2. Cell Counting Kit-8 Assay

Colon cancer cells were seeded in 96-well culture plates (30% confluence per well) (NEST, Wuxi, China) and incubated at 37 °C. After overnight adhesion, cells were treated with increasing doses of berberine (Selleck Chemical, Houston, TX, USA) for 24 h or 48 h. The cell viability was measured using the cell counting kit-8 (TargetMol, Shanghai, China) according to the instructions.

### 2.3. Protein Digestion and Peptide Purification

The colon cancer cells were treated with different concentrations (0 µM, 20 µM, 40 µM) of berberine for 48 h, and the control (0 µM) was added with the same amount of dimethyl sulfoxide (Solarbio, Beijing, China). After treatment, the medium was removed and cells were rinsed three times with cold phosphate buffered saline (PBS) (Solarbio, Peking, China). The cells were lysed directly with 8 M urea/1 M NH_4_HCO_3_ (Merck, Darmstadt, Germany) solution and sonicated on ice until the solutions became clear [19]. Samples were reduced by 5 mM dithiothreitol (Merck, Darmstadt, Germany) at 37 °C for 1 h, and then were alkylated by 15 mM iodoacetamide (Merck, Darmstadt, Germany) at room temperature away from light for 30 min. The reaction was terminated by 2.5 mM dithiothreitol at room temperature for 10 min. The sample solutions were diluted to 50% with deionized water and digested with sequencing grade trypsin (Promega, Madison, WI, USA); enzyme to protein, 1:100, *w*/*w*) at 37 °C for 2 h with shaking. The solutions were further diluted to 25% and digested with sequencing grade trypsin overnight at 37 °C with shaking. The digested samples were adjusted to pH < 2 by 50% trifluoroacetate (Merck, Darmstadt, Germany), and were centrifuged at 15,000 g for 10 min to remove cell residues. The peptides were desalted with hydrophile-lipophile balance column (Waters, Milford, MA, USA) and eluted by 60% acetonitrile (Merck, Darmstadt, Germany)/0.1% trifluoroacetate.

### 2.4. Liquid Chromatography-Tandem Mass Spectrometry Analysis

The liquid chromatography-tandem mass spectrometry (LC-MS/MS) analysis were performed on an Orbitrap Fusion Lumos mass spectrometer (Thermo Fisher Scientific, Waltham, MA, USA) coupled with an online EASY-nanoLC™ 1200 instrument (Thermo Fisher Scientific, Waltham, MA, USA). The method of parameter configuration was the same as that previously described [33]. Peptides were separated on a nanoViper PepMap100 C18 column (75 µm × 25 cm, Thermo Fisher Scientific, Waltham, MA, USA). The mobile phase consisted of 0.1% formic acid (Thermo Fisher Scientific, Waltham, MA, USA) in water (A) and 0.1% formic acid/80% acetonitrile (B) (Thermo Fisher Scientific, Waltham, MA, USA). The gradient profile was set as follows: 3–7% B for 2 min, 7–35% B for 83 min, 35–68% B for 20 min, 68–100% B for 10 min and equilibrated in 100% B for 15 min. Mass Spectrometry (MS) analysis was performed using an Orbitrap Fusion Lumos Mass spectrometer. The spray voltage was set at 2.3 kV. Orbitrap MS1 spectra (Automatic Gain Control 4 × 10^5^) were collected from 350–1800 m/z at a resolution of 60 K followed by data-dependent HCD MS/MS (resolution 15,000, collision energy 30%) using an isolation width of 1.6 m/z. A dynamic exclusion time was set to 45 s.

### 2.5. Database Search and Label-Free Quantitation

Mass spectrometric data were searched against the UniProt/SwissProt (http://www.uniprot.org, accessed on 10 May 2020) human proteome database (20341 proteins, downloaded on 30 December 2019) using MaxQuant (Department for Proteomics and Signal Transduction, Martinsried, Germany) (version 1.6.3.3) [34]. The precursor and fragment ion mass tolerance were set to 5 ppm and 20 ppm, respectively. The enzyme specificity was set to trypsin, and two missed cleavages were allowed. The minimum peptide length was set to 7 amino acids. Cysteine carbamidomethylation was set as fixed, and methionine oxidation and N-terminal acetylation were set as variable modifications. A maximum of 5 modifications per peptide was allowed. The false discovery rates (FDR) of both peptide and protein identification were set to 1% [35]. Uniform pre-established criteria were used for pairwise comparison, and retain peptide-spectrum matches (PSMs) ≥ 5 and minimum two peptides are required for each protein. The “Match between runs” based on the accurate m/z and mass spectra retention time was used with a minimum 0.7 match time window and minimum 20 alignment time window [36].

The normalization of label-free quantitation (LFQ) was performed based on the total intensities of all detected peaks in each liquid chromatography-mass spectrometry data [37]. The medium of normalized ratios from non-modified peptides were used for the protein quantitation. Data processing was performed using Perseus version 1.5.0.31. Contaminants and protein groups identified by a single peptide were filtered from the data set. FDR was calculated as the percentage of reverse database matches out of total forward and reverse matches. The LFQ intensities were log2 transformed to reduce the effect of outliers, and the missing values were replaced with random values taken from a median downshifted Gaussian distribution to simulate low abundance LFQ values.

### 2.6. Online Database Resource and Bioinformatics Analysis

The mutational gene information of 448 colon cancer patients were obtained from Genomic Data Commons Data Portal (https://portal.gdc.cancer.gov/, accessed on 10 May 2020) [38]. Mutation profiles of all colon cancer cells were obtained from the Catalogue of Somatic Mutations in Cancer (COSMIC) (https://cancer.sanger.ac.uk/cosmic/, accessed on 10 May 2020) [39]. Clustering analysis was calculated by the noise-robust soft clustering in “Mfuzz” R package. GO and KEGG pathway analyses were performed using Database for Annotation, Visualization and Integrated Discovery bioinformatic tools (https://david.ncifcrf.gov/, accessed on 12 May 2020) [40]. Reactome pathway analysis was performed by using the ClueGO plug-in and Cluepedia of Cytoscape software [41]. To display the multiple biological pathways involved by differentially expressed proteins, the PPI network was analyzed and performed by the Search tool for recurring instances of neighboring genes (STRING) (http://string-db.org/, accessed on 15 May 2020) and Cytoscape, with the interaction score ≥ 0.9 [42]. The transcriptional and translational levels of hub proteins in colon adenocarcinoma tissue were investigated by GEPIA (http://gepia.cancer-pku.cn/, accessed on 12 December 2020) and HPA (https://proteinatlas.org/, accessed on 15 December 2020) [43,44]. To validate potential therapy targets of colon cancer, the genetic dependencies in colon cancer cells were analyzed by DepMap datasets (https://depmap.org/portal/, accessed on 17 December 2020), in which genome-wide RNAi or CRISPR loss-of-function screens could be used to systematically identify essential genes across hundreds of human cancers [45,46].

## 3. Results

### 3.1. Berberine Inhibits Proliferation of Different Mutation Types of Colon Cancer Cells

**Figure 1:**
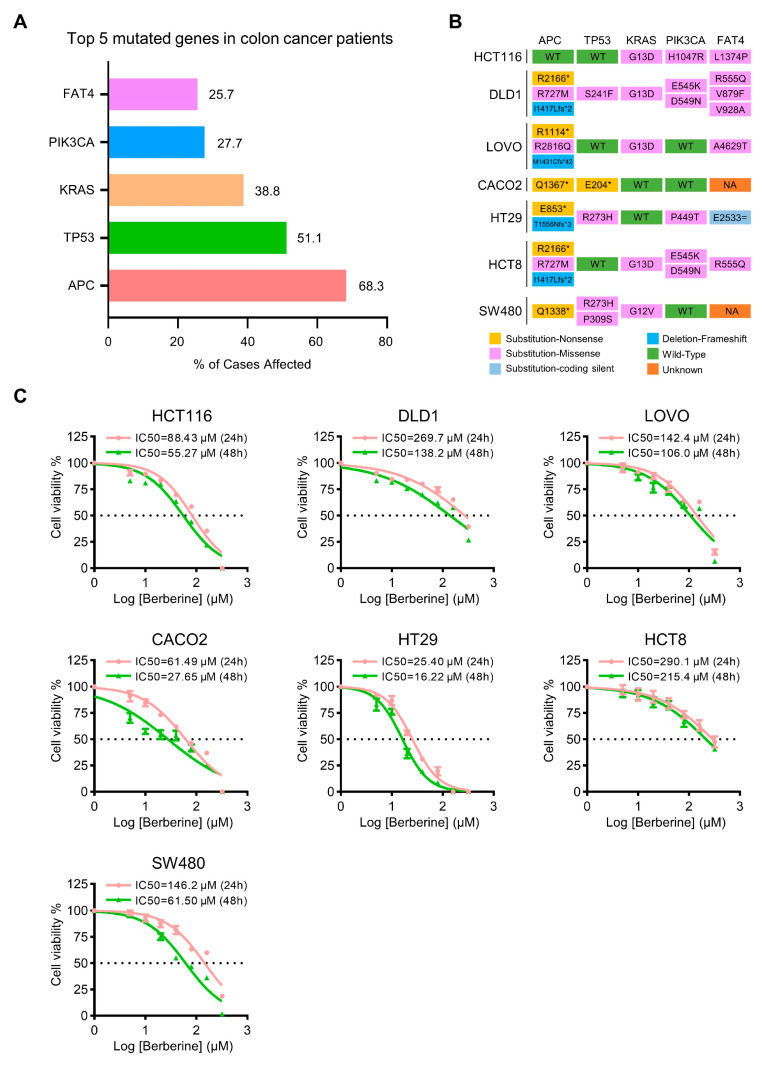
Anti-cancer effect of berberine on different mutation types of colon cancer cells. (**A**) Top five mutated genes in colon cancer patients. The data were obtained from Genomic Data Commons Data Portal [38]. The x-axis represents the percentage of cases in the cohort affected by each mutated gene; the y-axis lists the top five mutated genes in 448 colon cancer patients. (**B**) Mutational types and genetic abnormalities in seven commonly used colon cancer cell lines (HCT116, DLD1, LOVO, CACO2, HT29, HCT8, and SW480). The data were obtained from the Catalogue of Somatic Mutations in Cancer (COSMIC) [39]. Different mutational types and genetic abnormalities are represented in different colors. (**C**) Cell viabilities of colon cancer cell lines treated with increasing doses of berberine for 24 h or 48 h. The cell viability was assessed by CCK8. The data are presented as mean ± SD (*n* = 3). * *p* < 0.05.

Based on the mutation data from Genomic Data Commons Data Portal, the top five abnormally expressed or mutated genes in 448 colon cancer patients are APC, TP53, KRAS, PIK3CA, and FAT4. The abnormal expression or mutation of these five genes exist in most of colon cancer patients [38] (Figure 1A). To study the widely inhibitory effects of berberine on colon cancer cells, seven colon cancer cell lines with five major mutational genes, including HCT116, DLD1, LOVO, CACO2, HT29, HCT8, and SW480 cell lines, were chosen as cell models in vitro for this study (Figure 1B). Seven colon cancer cell lines were treated with different concentrations of berberine for 24 or 48 h, and cell viabilities were measured by cell counting kit-8. The results showed that berberine had a broad inhibition on all these different types of colon cancer cell lines in a concentration-dependent manner (Figure 1C), which is consistent with previous reports [27,28,29].

### 3.2. Proteomic Profiles of Colon Cancer Cells Treated with Berberine

**Figure 2:**
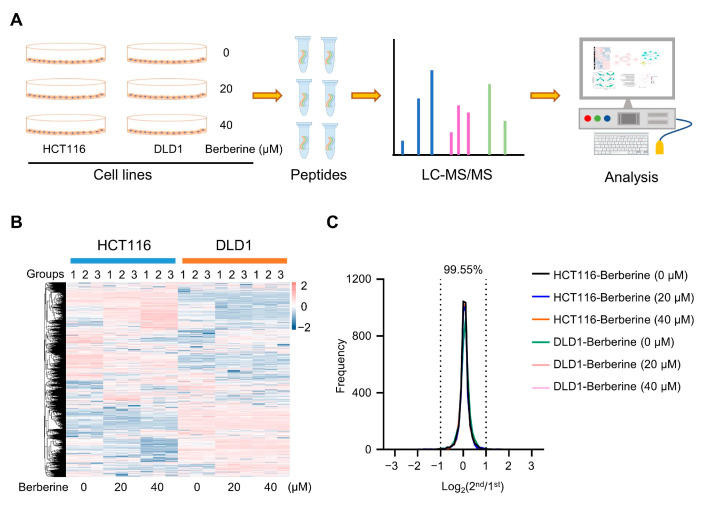
Proteome profiles and quantification of proteins among different berberine treatment conditions in HCT116 and DLD1 colon cancer cell lines. (**A**) Workflow of the proteomic study. Two colon cancer cell lines, HCT116 and DLD1, were separately treated with different concentrations of berberine for 48 h, and the control (0 µM) was added with same amount of dimethyl sulfoxide. The total proteins were then extracted, trypsin-digested, and underwent triplicate LC-MS/MS runs for label-free quantitative proteomics analysis. (**B**) Heatmap of quantified proteins in HCT116 and DLD1 cells. Cells were treated with different concentrations (0 µM, 20 µM, 40 µM) of berberine for 48 h. Groups indicate three technical repeats under each treatment condition; the color bar indicates the Z-score of intensities. (**C**) Ratio distributions of quantified proteins among technical replicates of each sample.

To identify the DEPs in the berberine-treated colon cancer cells, HCT116 and DLD1 cell lines were selected for analysis of label-free quantitative proteomics. The HCT116 cell line is a growth factor-independent cell line with the KRAS and PIK3CA mutation that has been shown to be invasive and highly motile in in vitro studies [47]. The DLD1 cell line contains all five major mutational genes, including APC, TP53, KRAS, PIK3CA, and FAT4. The two cell lines are representative in the study of colon cancer. In order to collect enough living cells, the cell viability of colon cancer cells was kept no less than 50% under selected berberine concentrations (0 µM, 20 µM, 40 µM). Cells were treated with different concentrations of berberine for 48 h, then, the samples were treated according to the workflow (Figure 2A). In HCT116 and DLD1 cells, a total of 5130 proteins were identified (Figure 2B, Table S1). The reproducibility of the experiments was evaluated by measuring the ratios between duplicate samples of the same group, which indicated that 99.55% of proteins varied within a two-fold change (Figure 2C). Therefore, a two-fold change in each direction was used as a reasonable cut-off for detecting protein alterations in the following analysis.

### 3.3. Analysis of DEPs in Berberine-Treated Colon Cancer Cells

**Figure 3:**
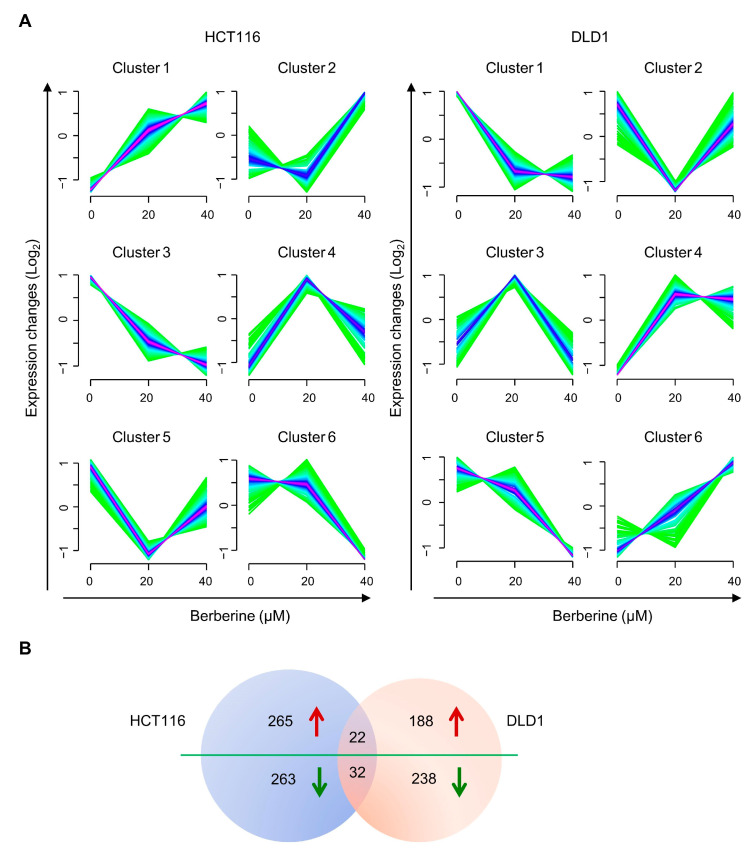
Soft clustering analysis of differently expressed proteins (DEPs) in berberine-treated HCT116 and DLD1 cells. (**A**) Protein expression changes of berberine-treated HCT116 and DLD1 cells in six clusters. The color varying from green to red represents that the degree of protein expressions matching with the patterns of the cluster. (**B**) Venn diagram of DEPs between HCT116 and DLD1 cell lines. Red arrows indicate up-regulated trend and green arrows indicate down-regulated trend.

The DEPs between the groups of 40 versus 20 μM, 20 versus 0 μM, and 40 versus 0 μM in both HCT116 and DLD1 cells were used for the noise-robust soft clustering analysis. Six clusters were obtained according to the expression modes of DEPs with different concentrations of berberine treatment (Figure 3A). A total of 865 DEPs were identified in HCT116 cells (Table S2). Since the protein expression values in clusters 1 and 2 showed a rising trend, the proteins in these two clusters were considered to be the up-regulated proteins. In contrast, clusters 3 and 6 showed a decreasing trend, and the proteins in the two clusters were considered to be the down-regulated proteins. Similarly, 675 DEPs were obtained in DLD1 cells (Table S2), and the protein expression values in clusters 4 and 6 showed a rising trend, while clusters 1 and 5 showed a decreasing trend. Meanwhile, the proteins in other clusters showed an inconsistent trend, so the proteins in these clusters were excluded from further analysis. In total, 287 up-regulated proteins and 295 down-regulated proteins were detected in HCT116 cells, and 210 up-regulated proteins and 270 down-regulated proteins were identified in DLD1 cells. There were 54 common DEPs in both cell lines, 22 of which were up-regulated and another 32 were down-regulated (Figure 3B, Table S3).

### 3.4. Functional Enrichment Analysis of Overlapping DEPs between HCT116 and DLD1 Cells

**Figure 4:**
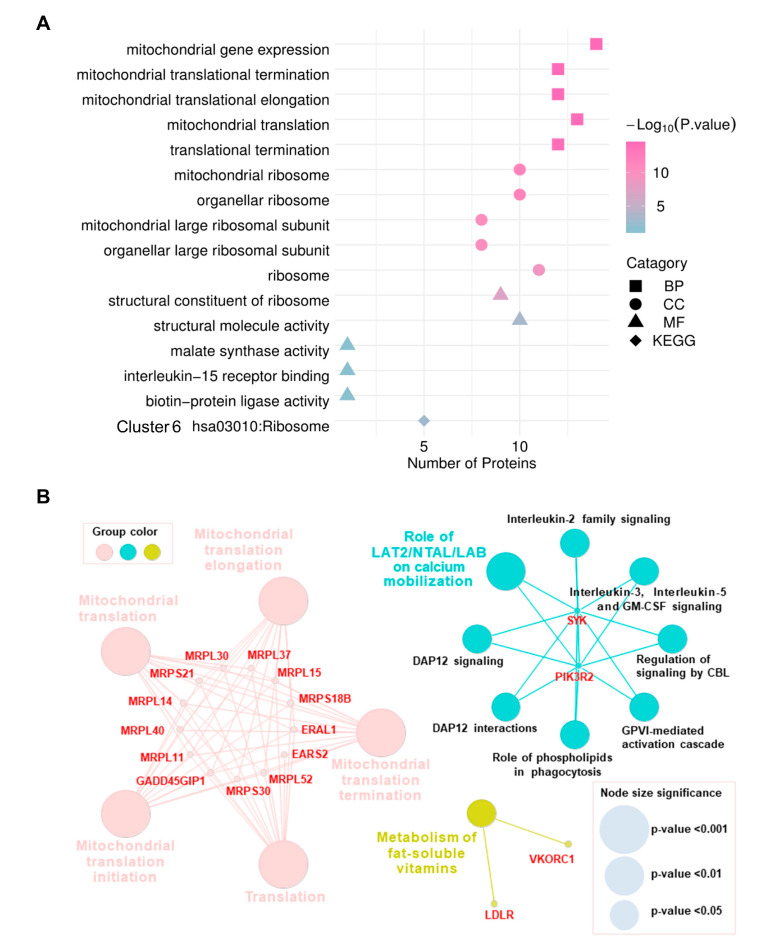
Functional enrichment analysis of common DEPs between HCT116 and DLD1 cells. (**A**) Gene Ontology (GO) and Kyoto Encyclopedia of Genes and Genomes (KEGG) pathway analysis of common DEPs between HCT116 and DLD1 cell lines. Analysis of categories include biological processes (BP), cellular component (CC), molecular function (MF), and Kyoto Encyclopedia of Genes and Genomes (KEGG). (**B**) Reactome pathway analysis of common DEPs between HCT116 and DLD1 cell lines; the gradual change of size from large to small stands for the *p*-values from low to high.

To explore the biological significances of common DEPs in both HCT116 and DLD1 cell lines, GO, KEGG and Reactome analysis were performed (Table S4). Regarding the biological processes (BP), the proteins were mainly involved in mitochondrial gene expression, mitochondrial translational termination, mitochondrial translational elongation, mitochondrial translation, and translational termination. For the cellular component (CC) category, proteins were localized in mitochondrial ribosome, organellar ribosome, mitochondrial large ribosomal subunit, organellar large ribosomal subunit, and ribosome. In addition, structural constituent of ribosome, structural molecule activity, malate synthase activity, interleukin-15 receptor binding, and biotin-protein ligase activity were the most terms of enrichment from molecular function (MF). In the KEGG enrichment analysis, it showed that these common DEPs were significantly enriched in ribosome (Figure 4A). The most specific Reactome pathways were associated with mitochondrial translation, role of LAT2/NTAL/LAB on calcium mobilization, and metabolism of fat-soluble vitamins (Figure 4B).

### 3.5. Selection of Hub Proteins in Berberine-Treated Colon Cancer Cells

**Figure 5:**
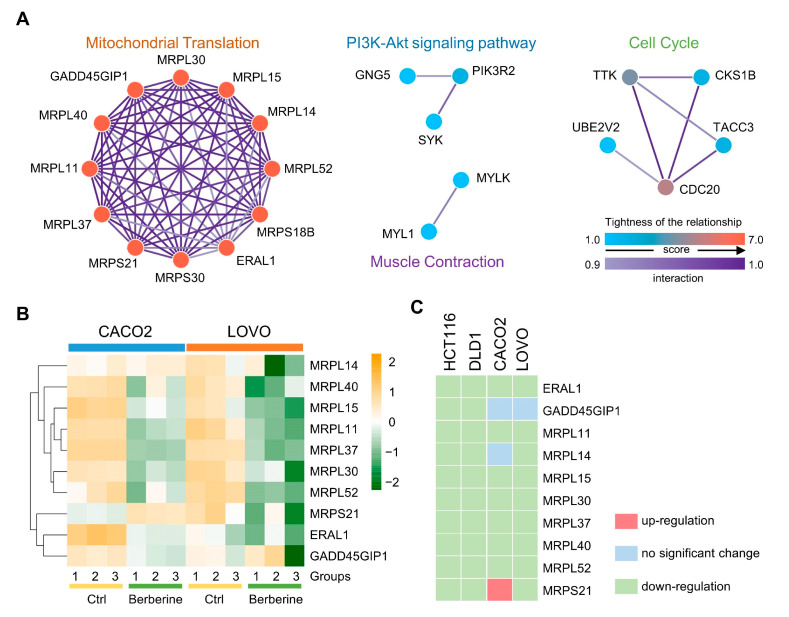
Selection of hub proteins in berberine-treated colon cancer cells. (**A**) Four clusters of Protein–Protein Interaction (PPI) networks from common DEPs between HCT116 and DLD1 cells. The color varying from blue to red represents the tightness of the relationship among DEPs; the deeper purple means a higher interaction score. (**B**) Heatmap of protein changes in berberine-treated CACO2 and LOVO cells. The data were obtained from re-analysis of previous reports [32]. Groups indicate three independent replicates used for LC-MS/MS analysis; color bar indicates the Z-score of intensities. (**C**) Ten proteins were commonly determined in four berberine-treated colon cancer cell lines, and trends of protein changes were indicated with three different colors.

To ascertain hub proteins in berberine-treated colon cancer cells, we investigated the interaction and physiological connections of common DEPs in both HCT116 and DLD1 cell lines. The PPI network was constructed and performed by STRING and Cytoscape (Figure 5A). Clusters of proteins in the PPI network were identified by the MCODE plugin in Cytoscape. Four significant clusters were selected and involved in mitochondrial translation, cell cycle, PI3K-Akt signaling pathway, and muscle contraction, respectively. The topology analysis for nodes in the PPI network showed that ERAL1, GADD45GIP1, MRPL11, MRPL14, MRPL15, MRPL30, MRPL37, MRPL40, MRPL52, MRPS18B, MRPS21, and MRPS30 were the core proteins with higher degree scores, and the 12 proteins were concentrated in the cluster of mitochondrial translation. The expression changes of 12 proteins were showed for berberine-treated HCT116 and DLD1 cells (Table 1).

To further verify the reliability of the proteins, we downloaded and re-analyzed berberine-treated CACO2 and LOVO colon cancer cells data from a published microarray-based proteomic study [32], and compared them with the 12 proteins identified in our study. The comparison results showed that 10 of 12 proteins identified in our study were also identified in the previous study. After re-analysis, expression changes of 10 proteins were showed for berberine-treated CACO2 and LOVO cells by a heatmap (Figure 5B, Table S5). To identify hub proteins, the protein changes of four berberine-treated colon cancer cell lines (HCT116, DLD1, CACO2 and LOVO) were presented by a heatmap, and 7 of 10 proteins were commonly down-regulated in all colon cancer cell lines (Figure 5C). Finally, the GTPase ERAL1, mitochondrial ribosomal proteins ERAL1, MRPL11, MRPL15, MRPL30, MRPL37, MRPL40, and MRPL52 were determined to be hub proteins with a commonly down-regulated trend in four berberine-treated colon cancer cell lines.

### 3.6. GTPase ERAL1 and Mitochondrial Ribosomal Proteins Including MRPL11, 15, 30, 37, 40, and 52 Are Predicted as Potential Targets of Berberine in Colon Cancer

**Figure 6:**
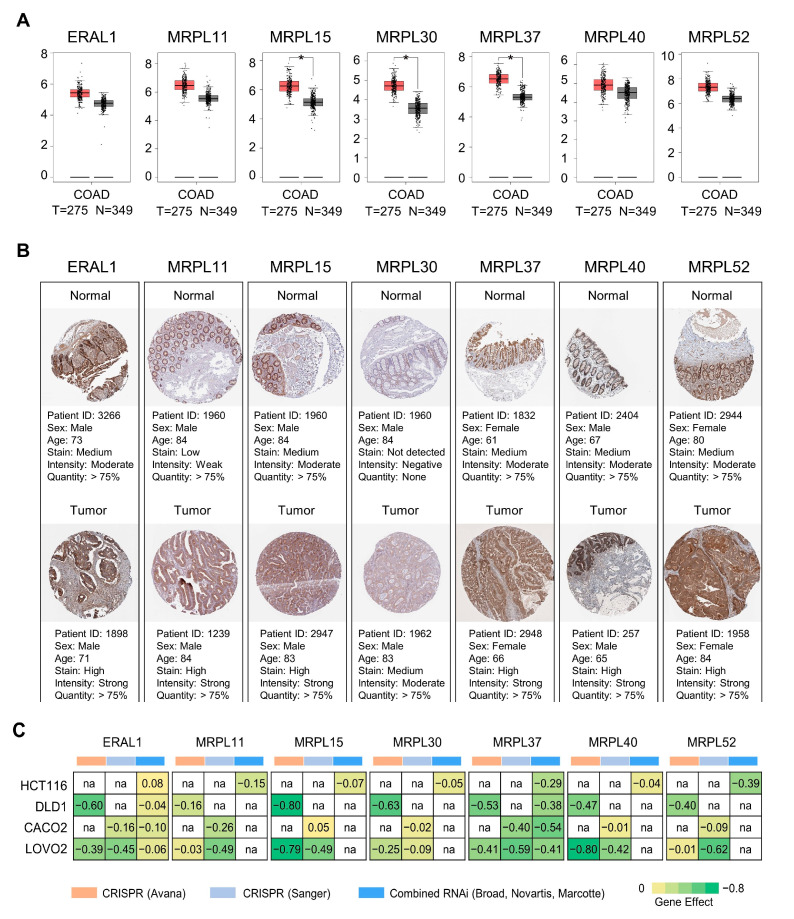
Validation of seven hub proteins as potential targets in berberine-treated colon cancer cells. (**A**) Validation of hub proteins in the transcriptional level by Gene Expression Profiling Interactive Analysis (GEPIA) [43]. T and N represents 275 tumor tissues and 349 normal tissues, respectively. * *p* < 0.05. (**B**) Validation of hub proteins in the translational level. Immunohistochemistry (IHC) staining data were obtained from the Human Protein Atlas (HPA) database, which demonstrated the expression status of hub proteins and the patient data [44]. (**C**) Dependency scores for seven hub proteins in four colon cancer cell lines from the Cancer Dependency Map (DepMap) [45,46]. Color groups indicates different sources of data; a lower score of gene effect means that a gene is more likely to be dependent on a given cell line; a score of 0 is equivalent to a gene that is not essential whereas a score of −1 corresponds to the median of all common essential genes.

Based on the transcriptome data from the GEPIA database [43], seven hub proteins identified in this study were over-expressed in colon tumors at the transcriptional level, and MRPL15, 30, and 37 were significantly over-expressed in colon tumor samples (a two-fold change with *p*-value < 0.05 as a cut off) (Figure 6A). In addition, the over-expressions of these seven hub proteins in colon tumors were also confirmed by using the immunohistochemistry (IHC) staining data from HPA database [44] (Figure 6B).

To validate the potential targets, genetic dependencies in four colon cancer cell lines were evaluated by CRISPR-Cas9 or RNAi, and related data were obtained from DepMap datasets [45,46] (Figure 6C). A higher genetic dependency means that the protein is more likely to be a potential target. For gene effect, a more negative number denotes a greater dependency in a given cell line, and minimum value in four colon cancer cell lines was selected as dependency score. The dependency scores of ERAL1, MRPL11, MRPL15, MRPL30, MRPL37, MRPL40, and MRPL52 for the four cell lines ranged from −0.8 to −0.49. These values indicated that silencing the genes expression of seven hub proteins by CRISPR-Cas9 or RNAi can significantly inhibit the proliferation of four colon cancer cells.

## 4. Discussion

Colon cancer is a common malignancy worldwide [1]. Thus, there is a requirement to develop more effective agents for the treatment of colon cancer. A number of studies showed that berberine as a biologic agent has great potential in the treatment of various cancer cells [48]. Berberine attenuates X-ray repair cross complementing 1 (XRCC1)-mediated base excision repair and sensitizes breast cancer cells to the chemotherapeutic drugs [49]. In HepG2 human hepatoma cells, berberine suppressed cyclin D1 expression, which inhibited cell proliferation [50]. A study showed that berberine induces apoptotic cell death via activation of caspase-3 and caspase-8 in HL-60 human leukemia cells [51]. Berberine has also been shown to inhibit melanoma cell proliferation and metastasis [52]. In addition, accumulating evidence indicates that berberine has multiple effects on colon cancer cells [20,27,28,53]. Although these studies provide important references for the study of berberine, the molecular targets and underlying mechanism of regulation remain unclear [30]. In this study, quantitative proteomics combined with online available online resources were used to systematically analyze the possible mechanism and targets of berberine for colon cancer treatment.

In our study, berberine was used to treat colon cancer cells with two different concentrations, and DEPs were clustered by the noise-robust soft clustering. The screened DEPs were classified into six clusters according to the different change trends of protein expressions. Then the clusters in response to berberine with a dose-dependent manner were screened, and all DEPs were derived from clusters with a decreasing trend or rising trend. Of note, when our manuscript was prepared, proteomics for berberine were reported in CACO2 and LOVO colorectal cancer cells by Tong et al., which further confirmed the anti-cancer potentials of berberine, and were complementary with our data [32]. However, a single medicinal concentration treatment for cellular response has its limitation in the research. In contrast, the analyzing method of soft clustering at different berberine concentrations used in this study could improve the reliability of the data.

Functional enrichment analysis revealed that common DEPs in HCT116 and DLD1 cells were mainly confined to mitochondrial proteins, a finding consistent with the previous reports of Tong et al. [32]. Mitochondria are bioenergetic, biosynthetic, and signaling organelles, and numerous studies have validated that mitochondria influences cancer initiation, growth, survival, and metastasis [54]. In addition, mitochondria bestow tumor cell bioenergies and oxidative stress to help them survive in the face of adverse environmental conditions [55]. Therefore, development of mitochondrial drugs will be important for the advance of cancer treatments. Moreover, the DEPs detected in both cells in our study were collectively associated with ribosomes. The ribosome is composed of numerous distinct proteins and nucleic acids and responsible for protein synthesis in living cells. Increased ribosome biogenesis and protein synthesis play essential roles in sustaining tumor cell growth and proliferation. Some recent studies showed that both increased numbers and altered modifications of ribosomes drive tumorigenesis [56,57]. Aberrant increases in nucleolar size and number reflect hyperactive ribosome biogenesis, which have been recognized as hallmarks of many cancers and associated with poor prognosis [58]. Therefore, inhibition of ribosome biogenesis represents a potential therapeutic avenue for cancer treatment.

Reactome enrichment analysis indicated that a portion of proteins were enriched in the role of LAT2/NTAL/LAB on calcium mobilization and metabolism of fat-soluble vitamins. Calcium is an essential nutrient for human health [59]. Previous research showed an approximately 70% lower risk of colon cancer comparing the highest to the lowest quartiles of calcium intake [60]. Additional studies have also demonstrated calcium could inhibit cell proliferation, and promote cell differentiation and apoptosis in colon cancer, which are likely mediated by extracellular calcium-sensing receptor (CaR) signaling [61,62]. Fat-soluble vitamins can play an important role in cancer prevention and treatment [63]. It has been extensively shown that vitamin D deficiency is associated with colon cancer, since active vitamin D metabolite inhibits proliferation and promotes epithelial differentiation of colon cancer by inhibiting the wnt/β-catenin signaling pathway [64,65,66]. Our study showed that berberine promotes calcium mobilization and metabolism of fat-soluble vitamins, which might be one of the possible mechanisms for its effects on colon cancer.

According to the results from the PPI network, some highly interacting proteins were found to be enriched in mitochondrial translation. We compared the changes of proteins in our study with data from Tong et al. [32], and found seven commonly down-regulated proteins (ERAL1, MRPL11, MRPL15, MRPL30, MRPL37, MRPL40, and MRPL52) from both studies. Furthermore, we found that these seven proteins have higher transcriptional and translational levels in tumor samples than in normal samples, and silencing their genes expression by RNAi or CRISPR-Cas9 can significantly inhibit the proliferation of colon cancer cells. Taken together, the evidence suggested that the seven hub proteins might be useful therapeutic targets in berberine-treated colon cancer cells.

Among these proteins, ERAL1 is a nuclear-encoded GTPase, which is localized in the mitochondrial matrix and associated with mitoribosomal proteins. Evidence showed that elimination of ERAL1 might lead to mitochondrial dysfunction and growth retardation [67,68], and knockdown of ERAL1 promotes reactive oxygen species generation that leads to autophagic vacuolization in HeLa cells [69]. These results suggest that ERAL1 is involved in cell viability. However, no studies have been performed on the functions of ERAL1 in colon cancer. MRPL11, MRPL15, MRPL30, MRPL37, MRPL40, and MRPL52 are mitochondrial ribosomal proteins that contribute to protein synthesis within the mitochondrion. Mitochondria are the key for virtually all facets of tumor progression [49], and they mediate aerobic energy conversion through the oxidative phosphorylation (OXPHOS) system. The recent evidence denoted the reliance of some cancer cells on OXPHOS [70]. Due to all components of the multimeric OXPHOS enzymes being synthesized in specialized mitochondrial ribosomes, targeting mitochondrial protein synthesis has led to new interventions to combat malignancies [71]. This evidence suggests that mitochondrial ribosomal proteins could be potential targets for cancer therapy. Interestingly, recent research demonstrated that high expression of MRPL52 might predict good survival in colorectal cancer [72]. We noted an opposite effect of this gene in our current study, namely that silencing MRPL52 could inhibit proliferation of different mutational colon cancer cells. This is probably due to the fact that tumor tissues in vivo are more complex than cell models in vitro. As for other proteins, no evidence has been reported on their functions in human colon cancers.

## 5. Conclusions

To summarize, by performing proteomic analysis on berberine-treated colon cancer cell lines, we showed that berberine could promote calcium mobilization and metabolism of fat-soluble vitamins. In addition, we found that berberine could impair mitochondrial function by inhibiting mitochondrial protein, and GTPase ERAL1 as well as mitochondrial ribosomal proteins including MRPL11, 15, 30, 37, 40, and 52 have potential to serve as candidate targets of berberine in colon cancer cells. Although direct and targeted experiments are still needed to validate the real functions of these proteins, the data presented here provide potential targets for the development of new therapeutic approaches toward colon cancer. Based on the anti-colon cancer potential of berberine, further research is encouraged to undertake larger randomized clinical trials in the strata of populations. Berberine as an adjuvant therapy or combine with other anti-colon cancer drugs may also open a new frontier for clinical treatment of colon cancer.

## Figures and Tables

**Table 1:** Expression changes of top 12 overlapping proteins in PPI degree scores were shown for berberine-treated HCT116 and DLD1 cells.

Protein Accession	Gene Name	Ratios in HCT116 (log_2_)		Ratios in DLD1 (log_2_)	
		20 vs. 0 μM	40 vs. 0 μM	20 vs. 0 μM	40 vs. 0 μM
O75616	ERAL1	−2.17	−2.20	−0.46	−1.75
Q8TAE8	GADD45GIP1	−0.08	−1.13	−1.17	−2.10
Q9Y3B7	MRPL11	−1.30	−2.00	−1.56	−2.63
Q6P1L8	MRPL14	−1.29	−1.23	−1.45	−2.79
Q9P015	MRPL15	−0.37	−1.07	−1.42	−2.34
Q8TCC3	MRPL30	−2.15	−2.42	−1.09	−1.82
Q9BZE1	MRPL37	−3.48	−4.17	−4.20	−3.55
Q9NQ50	MRPL40	0.19	−0.91	−0.44	−1.54
Q86TS9	MRPL52	−2.09	−1.85	−2.28	−2.00
Q9Y676	MRPS18B	−1.92	−2.06	−1.50	−2.12
P82921	MRPS21	−2.33	−3.07	−1.54	−1.02
Q9NP92	MRPS30	−2.33	−4.38	−2.83	−2.19

## Data Availability

The mass spectrometry data have been deposited to the ProteomeXchange Consortium (http://proteomecentral.proteomexchange.org, accessed on 15 June 2020) via the PRIDE partner repository with the dataset identifier PXD019749. (Account: reviewer91809@ebi.ac.uk; Password: 2ubralgl).

## Supplementary Materials

The following are available online at https://www.mdpi.com/2079-7737/10/3/250/s1, Table S1: All proteins identified from berberine-treated HCT116 and DLD1 cells, Table S2: Soft clustering analysis of DEPs in berberine-treated HCT116 and DLD1 cells, Table S3: Commonly identified DEPs in both berberine-treated HCT116 and DLD1 cell lines, Table S4: Bioinformatics analysis of overlapping DEPs between berberine-treated HCT116 and DLD1 cells, Table S5: Commonly determined ten proteins in two-sided studies. The list showed the protein changes in berberine-treated CACO2 and LOVO cells.

## Abbreviations

LC-MS/MS	Liquid chromatography-tandem mass spectrometry
MS	Mass Spectrometry
LFQ	Label-Free Quantitation
DEPs	Differently Expressed Proteins
GO	Gene Ontology
KEGG	Kyoto Encyclopedia of Genes and Genomes
PPI	Protein–Protein Interaction
BP	Biological Processes
CC	Cellular Component
MF	Molecular Function
DepMap	Cancer Dependency Map
